# Diagnostic Yield and Histopathological Features of Colorectal Lesions Detected Through a Regional Screening Program from the South-West Oltenia Region, Romania

**DOI:** 10.3390/cancers18111761

**Published:** 2026-05-28

**Authors:** Alexandra-Georgiana Bocioaga, Oana-Iulia Cretu, Alex Emilian Stepan, Raluca Niculina Ciurea, Cosmin Obleaga, Victor-Mihai Sacerdoțianu, Dan Nicolae Florescu, Dan Ionuţ Gheonea, Mirela-Marinela Florescu

**Affiliations:** 1 Research Center of Gastroenterology and Hepatology, University of Medicine and Pharmacy Craiova, 200349 Craiova, Romania; alexandra.bocioaga@umfcv.ro (A.-G.B.); mihai.sacerdotianu@umfcv.ro (V.-M.S.); dan.florescu@umfcv.ro (D.N.F.); dan.gheonea@umfcv.ro (D.I.G.); 2 Department of Biochemistry, University of Medicine and Pharmacy of Craiova, 200349 Craiova, Romania; 3 Department of Pathology, University of Medicine and Pharmacy of Craiova, 200349 Craiova, Romania; alex.stepan@umfcv.ro (A.E.S.); raluca.ciurea@umfcv.ro (R.N.C.); mirela.florescu@umfcv.ro (M.-M.F.); 4 Department of Surgery, University of Medicine and Pharmacy of Craiova, 200349 Craiova, Romania; cosmin.obleaga@umfcv.ro; 5 Department of Gastroenterology, University of Medicine and Pharmacy of Craiova, 200349 Craiova, Romania

**Keywords:** colorectal cancer screening, screening colonoscopy, colorectal polyps, histopathology

## Abstract

**Background/Objectives**: Colorectal cancer (CRC) represents a major cause of cancer-related morbidity and mortality worldwide, particularly in regions with delayed or incomplete implementation of population-based screening programs. Evidence describing the burden and characteristics of colorectal lesions detected through organized screening initiatives in Eastern Europe is still limited. **Methods**: This retrospective study analyzed data from a regional CRC screening program conducted between November 2020 and December 2023 in South-West Oltenia, Romania. A total of 1550 individuals aged 50–74 years underwent colonoscopic evaluation following fecal immunochemical testing or direct referral based on risk assessment. Lesion characteristics, including size, anatomical distribution, histopathological subtype, dysplasia grade, tumor differentiation, and resection margin status, were systematically analyzed. Associations between demographic variables and lesion characteristics were evaluated using appropriate statistical methods. **Results:** At least one colorectal lesion was identified in 63.7% of colonoscopies, with a total of 5001 lesions detected. Most lesions measured <10 mm. Serrated lesions (51.4%) and conventional adenomas (42.2%) predominated, while invasive adenocarcinoma was identified in 6.2% of cases. Increasing lesion size was significantly associated with higher-grade dysplasia, incomplete resection margins and advanced histopathological features (*p* < 0.05). Age showed significant associations with histopathological subtype and resection margin status, whereas male sex was linked to higher-grade dysplasia and adenocarcinoma (*p* < 0.05). **Conclusions**: This study revealed a relatively high prevalence of precancerous and malignant colorectal lesions in a previously unscreened population. The strong associations between lesion size, dysplasia severity, and resection completeness underscore the importance of early detection and quality-controlled colonoscopy. These findings support the expansion of organized CRC screening programs and the integration of quality indicators to enhance diagnostic accuracy.

## Simple Summary

Colorectal cancer remains a leading cause of death worldwide, particularly in areas where access to screening programs is limited, delaying early diagnosis and reducing opportunities for prevention. This study analyzed a regional colorectal cancer screening program in South-West Romania, identifying colorectal lesions in 63.7% of patients. Lesion size was significantly associated with dysplasia severity and advanced histopathological features, while age and sex influenced lesion type and aggressiveness. These findings highlight the need to expand screening programs and improve colonoscopy quality for early detection of colorectal cancer.

## 1. Introduction

From a global epidemiological perspective, the incidence of colorectal cancer (CRC) and the substantial burden of precancerous lesions fully justify the implementation of screening programs, particularly in developing countries, where such initiatives are absent or insufficiently structured. A global meta-analysis reported a prevalence of approximately 23.9% for adenomas and 4.6% for advanced adenomas among average-risk populations undergoing screening colonoscopy [1]. These findings suggest that, in the absence of organized screening, a substantial number of individuals harboring potentially malignant lesions remain undetected until more advanced stages.

Evidence from studies conducted across diverse populations has demonstrated that the prevalence of advanced neoplasia is not negligible even among individuals younger than 50 years. For instance, in a USA cohort, the prevalence of advanced neoplasia in the 45–49 year age group was approximately 5.0% [2]. This observation is particularly relevant for resource-limited countries, where the initiation of screening programs is often delayed and lesions are often detected at older ages, resulting in a higher proportion of advanced lesions at first clinical assessment. A key element in the pathway of progression toward colorectal cancer (CRC) is represented not only by conventional adenomas but also by serrated lesions—particularly Sessile Serrated Lesions (SSLs) and Traditional Serrated Adenomas (TSAs). These lesions tend to be subtle, are frequently located in the proximal colon, are more difficult to detect, and have been associated with a significantly increased risk of synchronous advanced neoplasia (odds ratio ~3.5) [3]. Consequently, comprehensive screening strategies must explicitly consider these lesion subtypes to achieve optimal effectiveness.

The histopathological classification of colorectal lesions is essential in the context of cancer prevention, as different lesion types exhibit distinct biological behavior and malignant potential. Conventional adenomas and serrated lesions represent the main precursors of colorectal cancer and follow different pathways of carcinogenesis. Therefore, the identification and characterization of these lesions during screening programs are critical for risk stratification and for guiding appropriate surveillance and therapeutic strategies aimed at preventing cancer development.

The evaluation of dysplasia grade in colorectal lesions is particularly important, as it reflects the severity of neoplastic transformation and the risk of progression to invasive carcinoma. Comparing dysplasia grades between sexes may provide insights into potential sex-related differences in tumor biology and risk factor exposure, thereby contributing to improved risk stratification and the development of more targeted screening and surveillance strategies.

In developing countries, the phenomenon known as the “first screening wave”—characterized by the detection of a substantial burden of polyps and advanced lesions during the initial colonoscopy—is commonly encountered in previously unscreened populations. The absence of prior screening contributes to a higher proportion of advanced lesions at first clinical presentation and, implicitly, to an increased risk of colorectal cancer. In our local study, which identified a considerable number of malignant polyps and precancerous lesions, these findings are consistent with observations reported in the international literature [4,5,6].

The implications for both clinical practice and public health policy are clear. Foremost, there is a need to implement a structured screening program that integrates detection modalities (such as fecal-based tests and colonoscopy) and targets age groups younger than those traditionally recommended, in accordance with recent evidence demonstrating the presence of advanced neoplasia in individuals under 50 years of age [1]. In addition, the quality of endoscopic practice must be systematically monitored, with performance indicators such as the adenoma detection rate (ADR) and the serrated lesion detection rate (SDR/SSL-DR) becoming essential metrics for quality assessment [7,8]. Finally, given the limited resources available in many countries, even partial screening coverage can yield a substantial reduction in colorectal cancer incidence through the early detection and resection of lesions with malignant potential.

The epidemiological profile of colorectal polyps is shaped by multiple factors, including age, sex, lifestyle patterns, dietary patterns, obesity, and tobacco use. These determinants are increasingly prevalent in socio-economically developing countries that are rapidly adopting a “Westernized” lifestyle without the concomitant development of comprehensive prevention and screening programs [3]. Consequently, the frequent detection of advanced lesions or polyps that have already undergone malignant transformation in unscreened populations can be regarded as an expected consequence of cumulative exposure to these risk factors combined with the absence of systematic colorectal evaluation through organized screening initiatives [1,2,3,8].

The histopathological characterization of colorectal lesions is essential in the context of cancer prevention, as different lesion subtypes and degrees of dysplasia are associated with distinct risks of progression to invasive carcinoma. Therefore, identifying these features during screening enables appropriate risk stratification, targeted surveillance, and timely therapeutic intervention, ultimately reducing colorectal cancer incidence.

In conclusion, our local findings from the South-West Oltenia region of Romania (Dolj, Gorj, Olt, Mehedinți, and Vâlcea), which reveal a high prevalence of malignant polyps and precancerous lesions at first clinical contact, underscore the urgent need to establish and expand colorectal cancer screening within the national context. Rigorous implementation of such programs, combined with continuous quality monitoring of endoscopic procedures and adaptation to local healthcare realities, has the potential to meaningfully reduce the burden of colorectal cancer and improve long-term patient outcomes.

The present study aimed to assess the diagnostic yield and histopathological characteristics of colorectal lesions detected through a regional screening program in a previously unscreened population from the South-West Oltenia region of Romania.

## 2. Materials and Methods

The prevention and early detection of colorectal cancer represent major health priorities. In November 2020, a pilot initiative entitled ‘Advanced Medical Services for the Prevention, Diagnosis, and Endoscopic Treatment of Colorectal Cancer’ was launched in Romania’s South-West Oltenia region. Developed under the coordination of the University of Medicine and Pharmacy of Craiova, the project aimed to enhance public awareness, provide education, and promote participation in the colorectal cancer screening program among residents of the Dolj, Gorj, Olt, Mehedinți, and Vâlcea counties. Within the framework of the screening program, all procedures, including fecal immunochemical testing (FIT) and colonoscopy, were provided free of charge, being covered by the European Union through the European Social Fund, within the Human Capital Operational Program 2014–2020, financial contract number: POCU/756/4/9/136442.

According to international guidelines, the prevention program targeted apparently healthy individuals aged 50 to 74 years at the time of enrollment, with particular emphasis on engaging a substantial number of participants from underserved or socioeconomically disadvantaged communities within the South-West Oltenia region. This project enrolled over 50,000 individuals, facilitating the identification of persons at increased risk for colorectal cancer.

Patients were identified based on lists supplied by participating general practitioners. All patients provided informed consent and completed a questionnaire designed to evaluate colorectal cancer risk, including personal symptoms and family history. Based on their responses, patients were either assigned to fecal immunochemical testing (FIT) using the SENTiFIT^®^ 270 analyzer (Sentinel Diagnostics, Milan, Italy) for the detection of occult gastrointestinal bleeding, corresponding to the average-risk population, while those identified as high-risk—defined by the presence of clinical symptoms suggestive of colorectal pathology, including rectal bleeding or a suspected rectal tumor detected during digital rectal examination—were referred directly for colonoscopic evaluation. Patients with a positive fecal immunochemical test (FIT) result (≥20 µg hemoglobin per gram of feces) were referred for lower gastrointestinal endoscopy, whereas those with negative results were advised to repeat the screening test biennially. All endoscopic procedures were performed under sedation and supervised by an anesthesiologist, except in patients with contraindications to anesthesia or those who explicitly declined sedation. Premalignant lesions identified during the procedure were removed endoscopically whenever feasible; in cases where complete removal was not achievable during the same session, patients were referred for further evaluation within the public healthcare system. Colonoscopy quality indicators were monitored, including withdrawal time (recorded from cecal intubation to scope removal), adenoma detection rate (ADR), defined as the proportion of procedures in which at least one conventional adenoma was identified, and cecal intubation rate (CIR), defined as the proportion of procedures in which the cecum was successfully reached, and bowel preparation quality assessed using the Boston Bowel Preparation Scale. When tumors or other lesions necessitating histopathological confirmation were detected, biopsy specimens were obtained and subsequently analyzed by experienced pathologists. All histopathological evaluations were performed by 2 experienced gastrointestinal pathologists. In cases of diagnostic uncertainty, a third pathologist reviewed the slides to reach consensus. The clinical and morphological parameters evaluated included patient age and sex, lesion subtype, resection technique, polyp dimensions, histopathological features, the presence and degree of dysplasia, tumor differentiation, and the status of the adenoma resection margins. Lesions were categorized according to the classification criteria proposed by the World Health Organization (WHO) in 2019 [9].

Collected tissue samples were fixed in 10% neutral buffered formalin and processed through routine paraffin embedding, followed by staining with Hematoxylin and Eosin (HE). Histological images were obtained with a Nikon Eclipse E600 microscope (Nikon Corporation, Tokyo, Japan) using Lucia 5 imaging software for acquisition and processing. Statistical analyses were conducted using IBM SPSS Statistics Faculty Packs, Version 26, Release 26.0.0.0 (IBM Corp, Armonk, NY, USA). Differences in continuous variables were assessed using the unpaired Student’s *t*-test, whereas categorical variables were analyzed with the Chi-square contingency test. A *p*-value < 0.05 was considered statistically significant. To account for clustering of multiple lesions within the same patient, multivariate logistic regression analysis was performed at the patient level using the most advanced lesion identified in each patient. The model evaluated the independent association of lesion size, age, and sex with advanced neoplasia.

Upon confirmation of colorectal cancer, patients underwent additional staging investigations and were referred to oncology and surgical departments for further management.

## 3. Results

Within the regional colorectal cancer screening program conducted between November 2020 and December 2023, a total of 51,437 (21,328 men and 30,109 women) individuals aged 50–74 years were invited to participate in the screening program. Based on predefined risk criteria, participants were categorized into medium- and high-risk groups. Those classified as medium risk were offered fecal immunochemical testing (FIT), whereas individuals in the high-risk group were directly referred for colonoscopic evaluation. Risk stratification was performed at enrollment using a structured questionnaire completed by participants together with their general practitioner. The questionnaire assessed the presence of rectal bleeding or obvious rectal tumor identified during digital rectal examination.

Regarding colonoscopy performance, quality indicators were analyzed across the entire cohort and demonstrated high procedural quality. The mean withdrawal time was 10.87 ± 4.23 min, exceeding the recommended minimum threshold of 6 min for adequate mucosal inspection. The adenoma detection rate (ADR) was 44.4% (688/1550). Cecal intubation was successfully achieved in 1479 out of 1550 colonoscopies, resulting in a cecal intubation rate of 95.42%. Data regarding specific exclusion criteria for CIR calculation, such as stenosing tumors or inadequate bowel preparation, were not systematically recorded; therefore, the rate was calculated using the total number of colonoscopies as the denominator. In addition, bowel preparation quality, assessed using the Boston Bowel Preparation Scale, demonstrated an overall mean score of 7.6 (2.33 ± 0.71 for the right colon, 2.54 ± 0.64 for the transverse colon, 2.71 ± 0.56 for the left colon), indicating overall adequate bowel cleansing. Overall, all quality indicators met ESGE standards for screening programs.

Among the screened population, 45,049 individuals completed FIT with valid results, of whom 2825 (6.27%) tested positive and were subsequently referred for colonoscopy. Among the positive cases, 1530 were male (54.16%), and 1646 originated from rural areas (58.27%). The mean age of the participants was 62.74 ± 6.80 years, reflecting a predominance of older individuals within the screening population. FIT values demonstrated substantial variability (mean: 267.80 ± 779.13 ng/mL), indicating an elevated risk of colorectal lesions among these patients. In addition, 88 individuals were identified as high-risk based on the risk assessment questionnaire and were directly referred for colonoscopic evaluation. Overall, 1550 participants underwent colonoscopy. Despite medical recommendations, 46.79% of eligible patients declined the procedure, underscoring the need for improved patient education and adherence strategies in colorectal cancer prevention. Of all colonoscopies performed, 988 examinations (63.74%) identified at least one polypoid or tumoral lesion, whereas 562 cases (36.26%) yielded no significant findings (Table 1).

Overall, 5001 lesions were detected, including polypoid, inflammatory, and tumoral lesions. However, detailed histopathological analysis was available for 1727 polypoid lesions, which were included in further analyses. Analysis of their anatomical distribution revealed that most lesions were located in the descending and sigmoid colon (1531 lesions; 30.61%). The anal canal represented the second-most frequent site, accounting for 1294 lesions (25.87%). Most anal canal findings represented non-neoplastic lesions and were therefore not included in the final histopathological analysis focused on colorectal precursor lesions. The ascending colon harbored 806 lesions (16.12%), followed by the transverse colon with 542 lesions (10.84%) and the rectum with 514 lesions (10.28%). The lowest lesion frequencies were observed in the cecum (297 lesions; 5.94%) and terminal ileum (17 lesions; 0.34%) (Table 1).

Among the patients included in the present analysis, lesions originating from male patients constituted the majority (64.7%, *n* = 1117), whereas the lesions from female patients represented 35.3% (*n* = 610), indicating a marked male predominance within the study cohort. Regarding age distribution among all analyzed polyps (*n* = 1727), participants ranged from 50 to 74 years, with a mean age of 63 years and a standard deviation of 9.9. Most individuals belonged to the age group >60 years (61.0%, *n* = 1053), whereas those aged between 50 and 60 years represented 39% (*n* = 674).

Across the entire cohort, 1727 polypoid lesions were analyzed, of which 887 (51.4%) were serrated polyps, 733 (42.2%) conventional adenomas, and 107 (6.2%) malignant polyps. This pattern reflects a predominance of serrated lesions, followed by conventional adenomas, while malignant polyps represented a relatively small fraction, suggesting a relatively lower burden of advanced neoplastic lesions in the study population (Table 2).

In terms of endoscopic management, most lesions were removed by cold snare polypectomy (59.4%), a method primarily applied to small and non-invasive lesions. Hot snare polypectomy with electrocautery was performed in 30.6% of cases, whereas simple biopsy was chosen in only 10% of procedures (Table 2).

The analysis of lesion size demonstrated that small polyps (<10 mm) constituted the vast majority of detected lesions, accounting for nearly 80% of all detected lesions. Polyps measuring 10–19 mm were observed in 14.5% of cases, while large lesions (>20 mm) were infrequently encountered, representing only 5.6% overall (Table 2).

**Figure 1:**
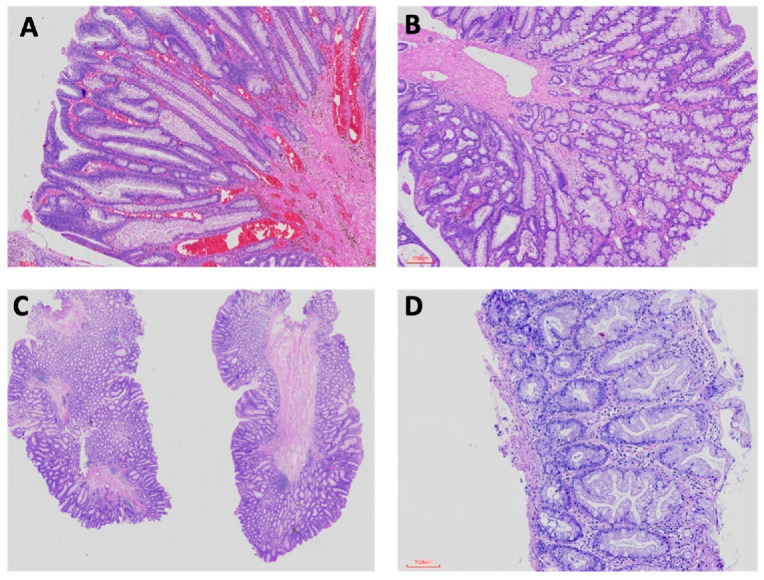
Histopathological aspects of colorectal polyps (HE staining). (**A**) Tubulovillous adenoma showing a mixed architectural pattern with both tubular and villous glandular structures supported by a fibrovascular stromal core; (**B**) Tubular adenoma characterized by closely packed tubular glands lined by dysplastic columnar epithelium; (**C**) Hyperplastic polyp showing elongated serrated crypts with preserved cellular architecture; (**D**) Sessile serrated lesion displaying distorted and dilated crypts with serrated architecture extending to the crypt base. Scale bar: (**A**,**B**) 300 μm; (**C**) 1000 μm; (**D**) 100 μm. HE: Hematoxylin–Eosin.

Histopathological evaluation demonstrated a considerable diversity of lesion types. Among hyperplastic polyps (HPs), 13.5% exhibited a microvesicular pattern and 2.4% a goblet cell-rich pattern. Serrated lesions included 144 sessile serrated lesions (SSLs) (8.3%) and 464 traditional serrated adenomas (TSAs) (26.9%). Within the group of conventional adenomas, the tubular subtype was predominant (47.9%), whereas tubulovillous adenomas were rare (0.9%). These findings highlight the predominance of tubular architecture and the exceptional occurrence of villous components in the analyzed population (Table 2, Figure 1).

**Figure 2:**
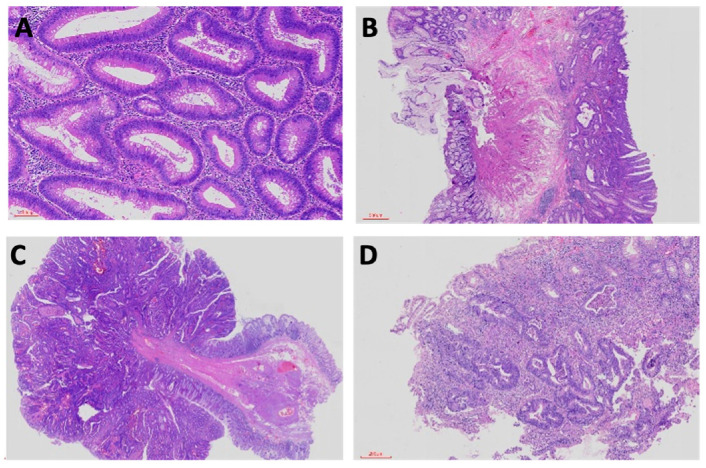
Histopathological aspects of colorectal dysplastic and neoplastic lesions (HE staining). (**A**) Low-grade dysplasia showing elongated crowded glands lined by dysplastic columnar epithelium; (**B**) High-grade dysplasia characterized by complex glandular architecture and marked epithelial atypia; (**C**) High-grade dysplasia with a free resection margin of more than 2 mm; (**D**) Adenocarcinoma showing infiltrative atypical glandular proliferation. Scale bar: (**A**) 100 μm; (**B**) 500 μm; (**C**) 200 μm; (**D**) 200 μm. HE: Hematoxylin–Eosin.

Assessment of dysplasia grade revealed that 23.9% of lesions showed no evidence of dysplastic changes. Low-grade dysplasia (LGD) represented the predominant histological category, identified in 48.9% of cases, followed by lesions exhibiting combined LGD and HGD features (9.8%) and high-grade dysplasia (HGD) (11.2%). Invasive malignant transformation, confirmed as adenocarcinoma, was present in 6.2% of the analyzed lesions (Table 2, Figure 2).

Regarding tumor differentiation, most lesions (72.8%) did not require grading, corresponding to non-invasive cases. Among lesions for whihc grading was applicable, moderately differentiated tumors (G2) were more frequently encountered (3.2%) compared to well-differentiated (G1) and poorly differentiated (G3) tumors. Carcinoma in situ was documented in 21% of cases (Table 2).

Evaluation of resection margins demonstrated that histopathological assessment was not feasible in more than 80% of specimens. Among evaluable cases, 14.2% exhibited negative margins exceeding 2 mm, while 3.4% showed negative margins at less than 2 mm, indicating an acceptable rate of complete excision in cases where histopathological margin evaluation was feasible (Table 2).

Age distribution by sex demonstrated that, in both females and males, the majority of patients belonged to the >60 year age group (360 females; 693 males), followed by those aged 50–60 years (250 females; 424 males). The relationship between sex and lesion size was evaluated across a total of 1727 lesions. Among female patients (*n* = 610), most lesions measured <10 mm (*n* = 496), followed by those measuring 10–19 mm (*n* = 79) and 20–29 mm (*n* = 28), while lesions ≥30 mm were infrequent (*n* = 7). A comparable pattern was observed in male patients (*n* = 1117), in whom lesions measuring 0–9 mm were predominant (*n* = 883), followed by those of 10–19 mm (*n* = 172) and 20–29 mm (*n* = 43); lesions ≥30 mm were identified in 19 cases. Analysis of histopathological subtype according to sex showed that, among female patients, tubular adenomas were the most prevalent lesions (*n* = 289), followed by traditional serrated adenomas (TSAs) (*n* = 155), sessile serrated lesions (SSLs) (*n* = 50), microvesicular hyperplastic polyps (*n* = 96), and goblet cell-rich hyperplastic polyps (*n* = 11), while tubulovillous adenomas were infrequent (*n* = 9). A similar pattern was observed in males, where tubular adenomas also predominated (*n* = 538), followed by TSAs (*n* = 309), SSL (*n* = 94), microvesicular hyperplastic polyps (*n* = 138), and goblet cell-rich hyperplastic polyps (*n* = 31); tubulovillous adenomas were identified in 7 cases. Statistical analysis did not reveal significant sex-related differences in histopathological subtype distribution, lesion size, or age category (*p* > 0.05).

**Figure 3:**
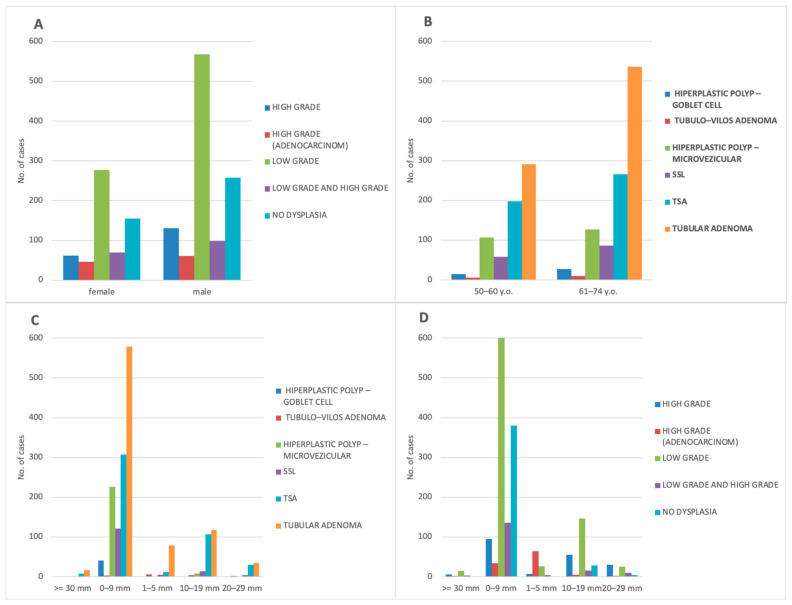
Distribution of colorectal lesions according to (**A**) patient sex and dysplasia grade, (**B**) patient age and histopathological type, (**C**) lesion size and histopathological type, and (**D**) lesion size and dysplasia grade. SSL: sessile serrated lesion; TSA: traditional serrated adenoma.

Assessment of the relationship between sex and dysplasia grade revealed notable quantitative differences. In women, low-grade dysplasia (LGD) was the most frequent finding (*n* = 277), followed by lesions without dysplasia (*n* = 155), mixed LGD–HGD lesions (*n* = 70), high-grade dysplasia (HGD) (*n* = 62), and adenocarcinoma (*n* = 46). In men, LGD remained the predominant category (*n* = 568); however, a higher number of lesions exhibiting HGD (*n* = 131) and adenocarcinoma (*n* = 61) was observed compared to females. This distribution reached statistical significance, confirming an association between sex and dysplasia grade (*p* = 0.047) (Figure 3).

In the subgroup of patients in the 50—60-year age group, small lesions (less than 10 mm fem) predominated (*n* = 527), followed by lesions measuring 10–19 mm (*n* = 103) and 20–29 mm (*n* = 30), while lesions ≥30 mm were observed in only 8 cases. A similar distribution was observed in patients older than 60 years, in whom lesions <10 mm remained predominant (*n* = 852), followed by those measuring 10–19 mm (*n* = 145) and 20–29 mm (*n* = 39); lesions ≥30 mm were uncommon (*n* = 17). Statistical analysis did not reveal a significant association between age and lesion size (*p* > 0.05). Analysis of the relationship between age and histopathological subtype demonstrated a differentiated distribution across the 1727 lesions included in the study. In the 50–60 years age group, tubular adenomas (*n* = 291) and traditional serrated adenomas (TSAs) (*n* = 197) were predominant. These lesion types were even more frequent in patients older than 60 years (536 tubular adenomas and 267 TSAs). Statistical analysis confirmed a significant association between age and histopathological subtype (*p* = 0.025) (Figure 3).

In the 50–60-year category, LGD remained the predominant finding (*n* = 319), followed by lesions without dysplasia (*n* = 166), mixed LGD–HGD lesions (*n* = 63), high-grade dysplasia (HGD) (*n* = 89), and adenocarcinoma (*n* = 39). In patients above 60 years, LGD continued to be the most frequent histological feature (*n* = 526), although an increase in the absolute number of HGD lesions (*n* = 106) and adenocarcinomas (*n* = 67) was observed. Regarding tumor differentiation, in the 50–60-year group, carcinoma in situ was reported in 150 cases, whereas differentiation grades G1 (*n* = 15), G2 (*n* = 20), and G3 (*n* = 5) were uncommon. Among patients older than 60 years, carcinoma in situ was the most frequent finding (*n* = 212), alongside sporadic cases of well-differentiated (*n* = 26), moderately differentiated (*n* = 35), and poorly differentiated tumors (*n* = 6). No statistically significant association was identified between age and tumor differentiation or dysplasia grade.

Assessment of resection margin status by age revealed that in the 50–60-year group, 576 cases had margins that could not be assessed, while 82 exhibited negative margins greater than 2 mm and 13 negative margins below 2 mm. In patients older than 60 years, negative margins exceeding 2 mm were more frequently observed (*n* = 161), along with 44 cases showing negative margins at less than 2 mm. The association between age and margin status was statistically significant (*p* = 0.003).

Cohort analysis demonstrated a statistically significant association between polyp size and resection margin status. Most cases in which margin assessment was not feasible corresponded to small polyps measuring 0–9 mm (*n* = 1210). Among polyps sized 10–19 mm, 76 cases exhibited negative margins greater than 2 mm, 11 had negative margins below 2 mm, while 164 were not evaluable. In the 20–29 mm category, 28 cases showed negative margins, 5 presented positive margins, and 38 could not be fully assessed. For polyps ≥30 mm, 14 cases had negative margins, 1 case had positive margins, and 11 were non-evaluable. The proportion of positive margins increased with lesion size, suggesting that larger polyps are associated with a higher risk of incomplete resection (*p* < 0.05).

Regarding the relationship between polyp size and histological subtype, distinct distribution patterns were observed. Hyperplastic polyps, particularly the microvesicular subtype, were almost exclusively identified in lesions smaller than 10 mm (*n* = 226). Sessile serrated lesions (SSLs) were most frequently encountered in small lesions (*n* = 126) but were also present in the 10–19 mm and 20–29 mm groups, indicating potential slow progression. Tubular and tubulovillous adenomas were predominantly observed in lesions exceeding 10 mm, whereas traditional serrated adenomas (TSAs) were mainly detected in the intermediate (10–19 mm) and larger (20–29 mm) size categories. A statistically significant association was identified between lesion size and histological subtype (*p* < 0.05) (Figure 3).

Distribution analysis according to lesion size demonstrated that, within the ≤9 mm group, most lesions were either non-dysplastic (*n* = 381) or exhibited low-grade dysplasia (LGD) (*n* = 558). As lesion size increased, a higher proportion of lesions with high-grade dysplasia (HGD) and HGD-associated adenocarcinoma was observed. For instance, in the 10–19 mm category, 55 HGD lesions and 5 HGD-associated adenocarcinomas were identified, whereas in lesions ≥30 mm, 6 and 2 cases were recorded, respectively. This pattern indicates a positive correlation between lesion size and dysplasia severity (*p* < 0.05) (Figure 3).

**Figure 4:**
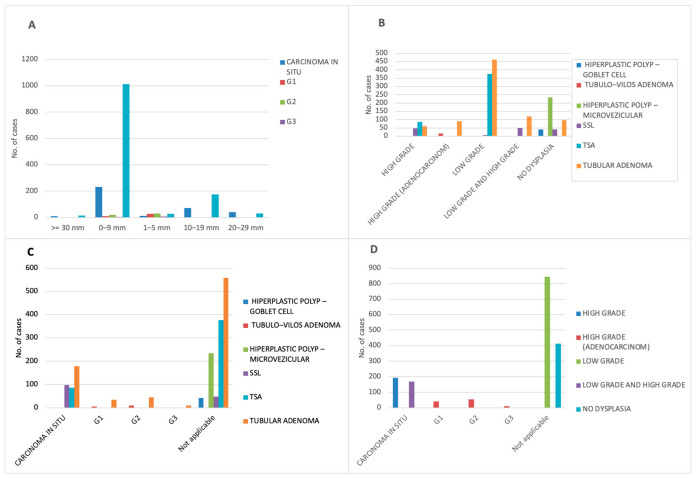
Distribution of colorectal lesions according to histopathological and morphological characteristics. (**A**) Lesion size and tumor differentiation grade. (**B**) Histological type and dysplasia grade. (**C**) Histological type and tumor differentiation grade. (**D**) Dysplasia grade and tumor differentiation grade. HP: hyperplastic polyp; SSL: sessile serrated lesion; TSA: traditional serrated adenoma; G1–G3: tumor differentiation grades.

Analysis of lesion size in relation to tumor differentiation revealed that carcinoma in situ predominated within the 0–9 mm group (*n* = 242), while lesions graded G1–G3 were more frequently associated with larger lesions. Specifically, two G2 lesions were observed in the ≥30 mm group, one G3 lesion in the 20–29 mm category, and three G1 lesions in the 10–19 mm category, indicating a statistically significant association (*p* < 0.05) (Figure 4).

Evaluation of the relationship between dysplasia grade and histological subtype showed a significant correlation between these variables. Hyperplastic polyps (microvesicular and goblet cell-rich) did not exhibit dysplasia (*n* = 276). Among sessile serrated lesions (SSLs), most were associated with high-grade dysplasia (*n* = 48) or mixed dysplasia (*n* = 49), while only 6 lesions demonstrated LGD. Traditional serrated adenomas (TSAs) predominantly displayed LGD (*n* = 377), with only one case progressing to HGD-associated adenocarcinoma. Tubular adenomas represented the largest category, with 462 LGD, 90 HGD, and 120 mixed dysplasia cases. Although less frequent, tubulovillous adenomas exhibited a more aggressive biological profile, with 16 HGD cases, including one invasive adenocarcinoma. The association between histological subtype and dysplasia grade was statistically significant (*p* < 0.05) (Figure 4).

Further analysis of histological subtype and tumor differentiation identified 1258 benign lesions without dysplastic changes, primarily consisting of microvesicular hyperplastic polyps (*n* = 234), goblet cell-rich hyperplastic polyps (*n* = 42), SSLs without dysplasia (*n* = 47), and TSAs without significant dysplasia (*n* = 377). For G1 differentiation, 41 cases were recorded, mainly tubular adenomas (*n* = 35) and tubulovillous adenomas (*n* = 5), along with one TSA case. G2 lesions included 45 tubular adenomas and 10 tubulovillous adenomas, while G3 lesions were exclusively identified in tubular (*n* = 10) and tubulovillous adenomas (*n* = 1). Carcinoma in situ was most frequently associated with tubular adenomas (*n* = 179), SSLs (*n* = 97), and TSAs (*n* = 86), showing a statistically significant association (*p* < 0.05) (Figure 4).

Tubular adenomas predominantly exhibited non-evaluable and negative resection margins ≥2 mm (n = 245). In the subgroup with margins negative <2 mm, tubular adenomas accounted for 51 cases, followed by tubulovillous adenomas (*n* = 8). These findings support a significant association between histological subtype and margin status (*p* < 0.05). To account for multiple lesions occurring within the same patient, multivariate logistic regression analysis was performed at the patient level using the most advanced lesion identified in each patient (*n* = 882). Lesion size remained significantly associated with advanced neoplasia, with lesions <10 mm showing lower odds of advanced histopathological features (OR = 0.208, *p* < 0.001), whereas age (*p* = 0.371) and sex (*p* = 0.533) were not independently associated with advanced histopathological findings.

## 4. Discussion

Colorectal cancer exhibits substantial geographic variability and an increasing incidence in socio-economically developing countries.

In a large-scale global meta-analysis conducted in 2020 and including more than 630,000 individuals, the prevalence of adenomas was estimated at approximately 23.9%, while adenomas with high-grade epithelial dysplasia accounted for about 4.6% and colorectal cancer for 0.4% of cases. These findings indicate that, in the absence of organized screening programs, a substantial proportion of the population already harbors lesions with malignant potential at the time of their first colonoscopy [2].

Numerous studies conducted in average-risk populations for colorectal cancer (CRC), including those in regions previously classified as “low-risk”, have demonstrated that the diagnostic yield of screening colonoscopy is far from negligible. Multiple reports from Iran, the Middle East, and various Asian countries have reported colorectal polyp prevalence rates ranging from 15% to 30%, with frequencies of adenomas and malignant adenomas comparable to—or even exceeding—those observed in Western countries [3,8,10,11,12]. In 2023, Ejtehadi F et al. conducted a study in an asymptomatic population, identifying through screening colonoscopy a spectrum of lesions ranging from adenomas with and without low-grade dysplasia to advanced adenomas and colorectal cancers. Based on these findings, the authors concluded that a colonoscopy-based screening strategy is justified and necessary even in regions traditionally regarded as low-risk [10]. More recent studies carried out in Asian and Eastern European populations further corroborate that, once colonoscopy is extended to previously unscreened populations, the proportion of neoplastic polyps and advanced lesions is unexpectedly high [10,11,12]. In this context, the high prevalence of malignant polyps and precancerous lesions observed in our cohort should be viewed not as an isolated finding, but rather as an indicator of a substantial latent disease burden within a population lacking an organized screening program.

Importantly, these findings have direct implications for colorectal cancer prevention. The high prevalence of precancerous lesions identified in this previously unscreened population (including conventional adenomas and serrated lesions) reflects a relatively high prevalence of lesions with malignant potential that could progress to colorectal cancer if left undetected. The detection and endoscopic removal of these lesions represent a key mechanism through which screening programs reduce colorectal cancer incidence and mortality.

Moreover, the observed associations between lesion size, dysplasia grade, and advanced histopathological features emphasize the critical role of early detection, as smaller lesions are more likely to be completely resected before malignant transformation occurs. These results support the role of organized screening programs not only as diagnostic tools but also as effective preventive interventions at the population level.

The collected data provide a comprehensive overview of the prevalence and distribution of colorectal lesions in this region.

Over the past decade, it has become clear that colorectal cancer (CRC) prevention is not limited to the detection of conventional adenomas, but also critically involves serrated lesions—most notably sessile serrated lesions (SSLs) and traditional serrated adenomas (TSAs)—which define the so-called serrated pathway of colorectal carcinogenesis. A meta-analysis focusing on SSLs reported a significant association between these lesions and an increased risk of synchronous advanced neoplasia, underscoring their role as true precursors of CRC rather than incidental endoscopic findings [13]. Furthermore, individuals diagnosed with serrated polyposis syndrome exhibit a substantially higher risk of CRC, particularly at the time of diagnosis, thereby justifying early identification and more intensive surveillance strategies [14]. Recent evidence from Asian populations indicates that the prevalence of SSLs may reach 5–6% of all colonoscopic examinations, with rates increasing with age and being higher among men. Moreover, several studies have reported that serrated lesions comprise between 3% and 20% of all detected colorectal polyps [15]. In the absence of structured screening programs and adequate clinical awareness of these entities, a significant proportion of colorectal cancers in the general population is likely to arise through this underrecognized serrated pathway [13,14,15].

The real effectiveness of colorectal cancer screening depends not only on whether colonoscopy is performed, but also on the quality of the examination. Beyond conventional quality indicators such as the adenoma detection rate (ADR), the proximal serrated polyp detection rate (PSPDR) has recently been proposed as an additional metric of endoscopic performance. Evidence from a population-based study demonstrated that each 1% increase in PSPDR was associated with an approximate 7% decrease in the risk of post-colonoscopy interval colorectal cancer, independent of ADR [16]. Furthermore, specific risk factors have been shown to be closely associated with the development of serrated polyps that are inherently difficult to detect [16,17,18,19,20]. In our cohort, the relatively high frequency of serrated polyps and advanced lesions lends support to the hypothesis that a substantial proportion of colorectal pathology remains effectively “hidden” until the first screening colonoscopy is performed [3,13,16,17].

Evidence from studies evaluating different types of colorectal polyps consistently shows that most polyps detected during colonoscopy are neoplastic, with a notable proportion qualifying as advanced adenomas. A recent study from China reported that nearly 77% of identified polyps were neoplastic, and approximately 44% of patients were found to have advanced adenomas [12]. Similarly, reports from Iran and Kurdistan, conducted in gastroenterology centers serving predominantly average-risk populations, have documented polyp prevalence rates of around 15–16%, with a substantial proportion of adenomas, serrated polyps, and even colorectal cancers identified at the time of the initial colonoscopic evaluation. When considered collectively, these findings indicate that in unscreened populations, the detection of a relatively large number of malignant polyps and precancerous lesions—such as those observed in our cohort—should not be interpreted as a localized anomaly. Rather, it reflects an endoscopic disease burden comparable to that reported in other developing countries or in healthcare systems where colorectal cancer screening is still evolving or insufficiently implemented [8,10,12].

Romania provides a representative example of a European country facing a substantial burden of colorectal cancer (CRC) and delayed adoption of population-based screening strategies. The South-West Oltenia region includes a significant rural population and has been reported to have disparities in access to healthcare services compared to national averages. Limited availability of specialized medical centers, lower health literacy, and disparities in healthcare coverage contribute to reduced participation in preventive programs such as colorectal cancer screening.

In Romania, access to healthcare services is primarily provided through the public health insurance system. However, inequalities in access to care have been described, particularly in underserved regions. These factors may contribute to delayed diagnosis and lower participation rates in screening programs. The ROCCAS pilot screening programs were launched only after 2019, alongside the establishment of a national methodological framework, a dedicated screening registry, and the corresponding digital infrastructure. These efforts were subsequently expanded through regional projects combining fecal immunochemical testing (FIT) with colonoscopic referral for individuals with positive results [21,22,23,24,25]. Preliminary data from these programs suggest that screening has the potential to reduce CRC-related mortality by up to 60%, while also highlighting several specific characteristics, including the central involvement of primary care physicians, significant interregional disparities, and the urgent need for targeted training of endoscopists and pathologists involved in screening pathways. Recent editorials have emphasized that the Romanian screening system is “prepared for nationwide implementation”; however, effective population coverage remains limited, leaving a significant proportion of eligible individuals without access to systematic CRC detection [23,24,25].

Across Europe, the 2003 Council of the European Union recommendations encouraged the development of organized colorectal cancer screening programs, together with those targeting breast and cervical cancer. The 2022 update reinforced this direction by calling for enhanced early detection strategies, broader target populations, and the adoption of modern screening technologies [26,27]. Despite these policy efforts, participation in screening and the utilization of colonoscopy remain highly heterogeneous among European countries. Recent studies have demonstrated significant disparities influenced by socioeconomic status, educational level, and structural differences in healthcare systems [28]. Romania, along with other Central and Eastern European states, remains in the phase of developing and extending colorectal screening programs, which largely explains the frequent identification of advanced lesions and relatively late-stage cancers during colonoscopies performed for symptomatic or opportunistic reasons in routine practice [21,22,23,24,25,28].

From the perspective of strategies aimed at improving colorectal cancer screening in resource-limited countries, our findings highlight the need for enhanced detection approaches. The literature supports several key directions, including optimization of triage algorithms by combining fecal-based tests with additional non-invasive biomarkers, which has been shown to improve the detection of advanced adenomas compared with the use of FIT as a standalone screening modality [29]. Another complementary strategy is the implementation of artificial intelligence (AI)-assisted technologies in colonoscopy. Although associated with higher upfront costs, these systems have demonstrated cost-effectiveness by increasing adenoma detection rates (ADR) and reducing the number of missed lesions, particularly small and flat polyps. In the context of our study, characterized by a high burden of lesions in a previously unscreened population, the gradual adoption of such technologies—initially in high-volume centers—may contribute to improving detection quality and reducing disparities in screening outcomes [28,29,30].

Several limitations of this study should be considered. The retrospective design may introduce biases related to data collection and completeness. In addition, the inclusion of individuals based on predefined risk criteria may have led to selection bias, as the study population was not restricted exclusively to asymptomatic average-risk subjects. The lack of follow-up data precluded the assessment of interval colorectal cancer. Furthermore, certain clinical and lifestyle variables, such as smoking status and body mass index, were not systematically recorded.

Despite these limitations, our study provides several relevant contributions. Beyond confirming known associations between lesion size, dysplasia severity and advanced histopathological features, our study contributes several relevant insights. First, it provides real-world evidence from a previously unscreened Eastern European population, a setting that remains underrepresented in the current literature. Within this context, the high prevalence of precancerous and malignant lesions observed in our cohort reflects a typical ‘first screening wave’ phenomenon, emphasizing a considerable latent disease burden in the absence of organized screening. Furthermore, our study illustrates a pragmatic screening model that combines FIT-based triage with direct colonoscopy referral for symptomatic individuals, which may be particularly applicable in resource-limited healthcare settings and may provide a useful framework for the progressive implementation and expansion of colorectal cancer screening programs in similar regions. In addition, the relatively increased frequency of serrated lesions observed in our cohort emphasizes the importance of recognizing this pathway in colorectal carcinogenesis.

Our results should be interpreted in the context of evolving colorectal cancer screening frameworks in Eastern Europe, where organized population-based programs are still being progressively implemented [21,25].

## 5. Study Limitations

Despite the relevance of our findings, several limitations should be acknowledged. The retrospective design of the study may have introduced biases related to data acquisition and recording. The inclusion of individuals selected based on predefined risk criteria may have introduced selection bias. The lack of follow-up data also precluded the assessment of interval colorectal cancer occurrence. In addition, important potential confounding factors, such as smoking status, body mass index, and medication history, were not systematically recorded.

Although patient-level regression analysis was performed using the most advanced lesion identified in each patient, residual bias related to the retrospective design and the presence of multiple lesions per patient cannot be completely excluded.

Another limitation of this study is the lack of detailed socioeconomic data, which prevented a stratified analysis of screening participation and lesion characteristics according to socioeconomic status. Although the area of residence (rural versus urban) was available, it may only partially reflect underlying disparities in access to healthcare. Future research should incorporate detailed socioeconomic variables to better evaluate inequalities in screening uptake and outcomes.

## 6. Conclusions

This study identified a relatively high prevalence of precancerous and malignant colorectal lesions in a previously unscreened population from South-West Romania. The detection of advanced conventional adenomas and high-grade dysplastic serrated lesions highlights the potential value of organized colorectal cancer screening and high-quality colonoscopic evaluation in identifying clinically relevant lesions at earlier stages.

Within the context of previous international studies, our findings support the potential importance of expanding colorectal cancer screening initiatives in developing countries and regions with limited screening coverage. However, given the retrospective design and regional nature of the present study, these findings should be interpreted cautiously and further validated through prospective population-based investigations [28,29,30].

The lack of screening programs promotes the progression of preneoplastic lesions to malignant tumors, often necessitating complex and costly surgical interventions. Within this framework, the early and accurate detection of polyps with malignant potential is essential for colorectal cancer prevention, contributing to the optimization of healthcare resource utilization and the reduction in morbidity and mortality rates [31].

## Figures and Tables

**Table 1:** General characteristics of the screened population.

Parameter		No.	%
Lesions	Present	988	63.74
	Absent	562	36.26
Segment	Descending/Sigmoid colon	1531	30.61
	Anus	1294	25.87
	Ascending colon	806	16.12
	Transverse colon	542	10.84
	Rectum	514	10.28
	Cecum	297	5.94
	Terminal ileum	17	0.34
Gender	Male	1117	64.7
	Female	610	35.3
Age (years)	50–60	674	39
	>60	1053	61

**Table 2:** Clinicopathological Features of Colorectal Polyps.

Parameter		No.	%
Lesion type	Conventional adenoma	733	42.2
	Malignant polyp	107	6.2
	Serrated polyp	887	51.4
Resection type	Biopsy	173	10
	Cold mucosectomy	1025	59.4
	Hot mucosectomy (electrocoagulation)	529	30.6
Size (mm)	0–9	1379	79.8
	10–19	251	14.5
	20–29	71	4.1
	≥30	26	1.5
Histopathological appearance	HP—goblet cell	42	2.4
	HP—microvesicular	234	13.5
	SSL	144	8.3
	TSA	464	26.9
	Tubular adenoma	827	47.9
	Tubulovillous adenoma	16	0.9
Dysplasia	No dysplasia	413	23.9
	LG	845	48.9
	LG and HG	169	9.8
	HG	193	11.2
	HG (adenocarcinoma)	107	6.2
Differentiation degree	Not applicable	1258	72.8
	G1	41	2.4
	G2	55	3.2
	G3	11	0.6
	In situ carcinoma	362	21
Adenoma Resection Margin	Cannot be assessed	1423	82.4
	Negative ≥ 2 mm	245	14.2
	Negative < 2 mm	59	3.4

HP, hyperplastic polyp; SSL, sessile serrated lesion; TSA, traditional serrated adenoma; LG, low-grade; HG, high-grade; G1, well-differentiated; G2, moderately differentiated; G3, poorly differentiated.

## Data Availability

The data presented in this study are available on reasonable request from the corresponding author. The data are not publicly available due to ethical restrictions, patient confidentiality, and GDPR regulations. Access to the data may be granted upon reasonable request and with permission from the relevant institutional ethics committee.

## Abbreviations

The following abbreviations are used in this manuscript:

ADR	Adenoma detection rate
AI	Artificial intelligence
CRC	Colorectal cancer
FIT	Fecal immunochemical test
HE	Hematoxylin and Eosin
HGD/HG	High-grade dysplasia
HPs	Hyperplastic polyps
LGD/LG	Low-grade dysplasia
PSPDR	Proximal serrated polyp detection rate
SDR/SSL-DR	Serrated lesion detection rate
SSLs/SSL	Sessile serrated lesions
TSAs/TSA	Traditional Serrated Adenomas
U.S.A	United States of America
WHO	World Health Organization
